# Supplementary material for: Harvesting and amplifying gene cassettes confers cross-resistance to critically important antibiotics
Source: PLoS Pathog. 2024 Jun 6;20(6):e1012235. doi: 10.1371/journal.ppat.1012235 (PMC11156391; doi:10.1371/journal.ppat.1012235)
Supplement: Attachment — Submitted filename: Dulyayangkul et al., 2023 V1 Referee Rebuttal.docx [file ppat.1012235.s002.docx]

Dear Editors,

We are grateful for the efforts of the referees in their thorough appraisal of our manuscript. We have revised the manuscript based on these comments, and have provided a detailed response to referees below.

Reviewer's Responses to Questions

Part I - Summary

Reviewer #1: This manuscript describes the characterization of an integron-base gene cassette that is present as a transposon flanked by IS26 elements. The work is clearly presented, but is missing key experiments. In addition, there are no page or line numbers in the manuscript which makes it difficult to point out changes. All page references below will be based on the PDF available to this reviewer.

We have added page and line numbers to the revised manuscript.

Reviewer #2: The authors offer a succinct and informative research piece detailing the mechanisms underlying pip/taz and amikacin resistance in Enterobacterales carrying AAC(6')-Ib-cr and OXA-1. While the identification of the IS6 transposition carrying these genes may not be novel, the demonstration of IS6-mediated increased expression and amplification of the transposon presents novel findings, specifically, in the context of amikacin and pip/taz resistance. I enjoyed reading this study.

We are grateful for this positive comment.

Reviewer #3: *** I have never peer reviewed using the part 1, 2 and 3 model. The intention of grouping into 2 and 3 was to suggest areas where the suggested change is stronger than stylistic but not to inflate the number of type 2 comments to cause a rejection ***

Carbapenem sparing options for therapy remain important notwithstanding the introduction of carbapenemase-inhibitors. This is especially true in resource limited settings and in countries without any marketed carbapenemase-inhibitors.

The major challenge with using PIPERACILLIN-TAZOBACTAM as such a strategy comes from the MERINO trial finding of "not non inferior". There are several reasons why that result may have occurred, and one of them is the inability of conventional testing to have properly inferred PIP-TAZO susceptibility (particularly in OXA-1).

On that background, the idea of using amikacin non-susceptibility to infer the potential presence of OXA-1 is intriguing and the methods used have shown the mechanism of why this should be considered and investigated.

I do worry a bit about some residual misclassification of amikacin and pip-tazo susceptibility because I believe that the Vitek cards (and EUCAST disc diffusion) can make errors, particularly when the organism is CEFTRIAXONE resistant and the "pretest probability" of resistance is higher. I think this needs to be acknowledged somewhere as a limitation. I don't think it invalidates the work -- after all, clinical labs are using these methods today and clinicians are acting upon them.

Because of known issues with Vitek 2 performance for TZP, our local diagnostic labs report Vitek AND disc susceptibility testing results for TZP. We have not noted a problem with Vitek reporting amikacin susceptibility (this may change if breakpoints change – see your comment later).

We do agree that since OXA-1 and AAC(6’)-Ib-cr are clustered in CTX-M-15 producers it is in 3GC-R isolates that Vitek TZP “issue” and so the benefit of our findings also cluster. However, we do report here a number of 3GC susceptible E. coli carrying OXA-1 and AAC(6’)-Ib-cr and there is the same degree of TZP “uncertainty” for these isolates (note our comment below about the minimal effect of CTX-M-15 on TZP MIC).

We absolutely agree that the proposed use of AMK to help improve sensitivity for TZP needs a lot of validation. However, the work reported here is primarily to explain the biology (given the remit of the journal) and our investigation of the potential clinical “benefit” of this finding must wait.

Part II – Major Issues: Key Experiments Required for Acceptance

Reviewer #1: 1. Pg. 11: has the location of the aac(6)-Ib-cr promoter been experimentally verified? If so, this should be referenced here. If not, how do the authors know that the deletion encompassed this region or that no mutations were in the promoter region in amikacin sensitive isolates.

The position of the hybrid promoter has now been confirmed as per your point 3. It is exactly where it was predicted to be based on the *E. coli* promoter consensus. This result has now been added to the text and into figure 2. At this point in the text however, we acknowledge that the promoter has not been defined and so we have removed mention of the promoter at this point in the manuscript. We then add this observation later in the text.

2. Pg 12: Is the statement “In all sequenced aac(6’)-Ib-cr-positive BSI E. coli in our survey, a copy of the insertion sequence IS26 was found upstream of aac(6’)-Ib-cr” referring to amikacin resistant isolates? If so, please clarify.

We are referring to all sequenced *aac(6’)-Ib-cr*-positive BSI *E. coli* in our survey. So that would include those which are susceptible and resistant.

3. The existence of a hybrid promoter conferred by the IS26 should be experimentally verified by 5’-RACE or primer extension analysis. Without this information, the single bp change in the proposed -10 region may not even be in a promoter region and may be altering gene expression in a post-transcriptional manner. In addition, the effects of the single base substitution on aac transcript levels should be experimentally verified by qRT-PCR analysis.

We have defined the position of the promoter using 5’RACE – result added to figure 2. The referee makes an interesting point about the possible existence of post-transcriptional control, however, we would remind them that the promoter is driving expression of a polycistronic mRNA carrying two ribosome binding sites (these are tandem gene cassettes and the biology of integron gene cassette expression is well established), and we have shown using proteomics that both encoded proteins are upregulated when the promoter is mutated to a sequence that is obviously closer to the well-known *E. coli* consensus. Hence there is no biologically plausible post-transcriptional explanation for this finding that could be resolved using qRT-PCR.

4. The authors proposed that an IS mediated duplication increased copy number of the aac gene. What were the endpoints of the amplified region? This information should be provided. Also, the specific copy number of genes within the amplified region should be determined by qPCR analysis.

We acknowledge that the wording suggested us favouring the preposition that IS-mediated amplification was “the mechanism” explaining why some isolates carried the genes at higher copy number than others. This was not what we meant. All that we have observed is increased copy number in some isolates. This could be due to the transposon being present on different plasmids in different isolates, each with a different copy number, or it could be due to IS-mediated amplification, or a mixture of both. We have therefore modified the text accordingly to make it clear that amplification is one possibility but the biology is being driven by copy number in any event.

We cannot define the “endpoint of the amplified region” in any of the isolates since to do that we would need to have a reference genome where the transposon is in an identical genomic location to the isolate from which the sequence reads have been obtained. (note we would need to do this for >60 isolates). Since it is a transposon, it can be in many (and often multiple) places in the genome. Hence all you see if you map reads from an isolate against a reference is the amplified region ending at the distal ends of each IS26. This would obviously support transposon amplification as the mechanism, but that could be an artifact of the read mapping method. For example, if the transposon were on a high copy plasmid in the sequenced isolate, one would then fail to see the copy number elevation spreading out beyond the end of the transposon because the reference locus would not include these plasmid genes. Similarly, it is not possible to use the assembly of the sequenced genome as a reference and then map the sequence reads to that because the fact that IS26 is present at multiple sites means that the reads are dumped in one place, truncating the assembly and meaning that the transposon (minus the entire IS26 transposase gene) is found in its own contig.

We would suggest that read mapping is perfectly adequate as a molecular counter, given it’s use in RNAseq without the need for low-tech validation, and since we have >60 isolates where the copy number (for two genes) has been measured, we cannot justify performing qPCR on them all. Particularly since the correlations presented in the paper (most notably between the two genes in the transposon) are so clear. And moreover that we have now added in data from Jackson et al. – see later.

Reviewer #2: I find no major issues with this work

Reviewer #3: Introduction:

I can tell where you are trying to take the first section of the introduction -- and I agree, that losing AG, ceftriaxone, and pip-tazo potentially leaves us little choice (especially since quinolone resistance is also frequently found in ARO with CTX-M). I just think this could benefit from some further reworking from the clinical context if the argument being advanced is clinical.

See note above, we do not want to make this paper too clinical given the remit of this journal. But we have added this “carbapenem-sparing angle to the modified manuscript.

Results:

“We took this as evidence that aac(6’)-Ibcr is not always a determinant of amikacin resistance.” This assumes that the phenotypic test for susceptibility was correct (and that the breakpoints are correct). To cross reference with methods.

*** NB in 2023 CLSI has updated Amikacin to be a breakpoint of <=4 for susceptibility based on achievable drug levels in humans vs. toxicity. I wonder if/when EUCAST will follow. And 4 may not be aggressive enough where predicted target attainment is as poor as 20% at 4. See:

https://academic.oup.com/ofid/article/7/7/ofaa084/5804675

This doesn’t invalidate what you are presenting, but would it change your 20% susceptible? Would it also change the “specificity” of the phenotype of amikacin R predicting OXA-1 if Amikacin S is limited to 4 instead of 8. Worth exploring?

We have added a clarification as requested that all our observations are dependent on the correct definition of “resistance”. Just so that those reading from the future are not confused.

We agree that the recent reduction in breakpoint since would likely reduce the ability for amikacin S/R to “predict” TZP S/R. One could still use a higher amikacin “breakpoint” to help “ratify” the TZP, whilst still using the “correct” breakpoint to define amikacin susceptibility itself. Notes added in discussion to this effect.

Discussion:

Reference 35 is not a randomized controlled trial -- it is mislabelled in pubmed. It is a retrospective cohort study.

Modified to clarify

Methods

The challenge with pip-tazo disc diffusion by EUCAST is that it can misclassify (see Henderson's MERINO paper in CID comparing to BMD). Would be worth adding this limitation (recognizing that most clinical labs do NOT do BMD).

Added as requested, though see note below.

How well do vitek MICs correlate with MICs from BMD? The tables should indicate you are using Vitek MICs. I am unsure if the Vitek has been validated for the 2023 CLSI amikacin breakpoints. Which vitek card(s) were used? Different cards may have different operating characteristics. Did that card have pip-tazo? If yes, why use disc as truth vs vitek? I agree with the decision but it should be justified.

Our diagnostic lab does not record Vitek “MICs” so we cannot say and we are only reporting the flag. The Vitek card (which includes TZP) has been added to the methods (this was done on isolation in 2000 so will be based on breakpoints and SOPs at that time). The diagnostic lab reports TZP Vitek AND disc S/NS flags because of widespread disagreement between these methods. For all other agents only the Vitek result is reported. We have performed an analysis comparing Vitek vs disc vs BMD across a range of isolates for TZP, though only a few of the isolates from this study (because there are other relevant mechanisms) and in our experience disc testing is more accurate than Vitek, particularly where the mechanism is OXA-1, which is why we used the disc testing result here. We have added some clarification in the methods but not the data as it relates to another piece of work.

Note also our point above that this paper is mainly about reporting on the biology. The proteomics data really show the effect more clearly than the AST, which is partly because of the inherent inaccuracy of AST. Even BMD using doubling dilutions.

Table 1 - please make clear the method of MIC.

This was BMD – now clarified

Part III – Minor Issues: Editorial and Data Presentation Modifications

Reviewer #1: 1. Introduction, page 8: E. coli should be Escherichia coli for the initial description.

The first use of this species name is in full – line 69.

2. Introduction, page 9 top: exists should be exist.

Modified as requested.

3. Fig. 3 Legend: More detail should be provided. For example, what does “mutant promoter” indicate?

Modified as requested

Reviewer #2: in paragraph one of the introduction, I think that the last sentence could be rewitten to remove "etc" from the list.

Modified as requested

the authors conducted a blastn screening of the transposon against genomes available on NCBI, revealing its presence in 714 genomes. Three questions arise from this screening: (1) Was the screen confined solely to the Enterobacterales?

No – we searched the whole database, modified in the text.

(2) Were specific thresholds (%ID, %COV, score) applied to confirm the presence or absence of the transposon?

We required 100% coverage and 100% identity, modified in the text

(3) Did the blast screen include only "complete" genomes, or were draft genomes also considered? If the latter, were the criteria for confirming presence/absence consistent across draft and complete genomes?

Only complete genomes, modified in the text. However, we have been able to screen one collection of raw sequence data, and from a previous survey in the US (Jackson et al), and we have provided our analysis, which adds considerable additional support to our findings.

Regarding the assertion that the transposon is "widely and globally demonstrated," can the authors confirm whether the 714 carriers of the transposon indeed represent a globally distributed collection? Have they ensured that this collection excludes repeated strains from outbreaks or single individuals? It is recognized that such data may not be universally available.

We acknowledge this point have removed the term, “globally” throughout. It is not possible to exclude outbreaks, but we hope the referee would acknowledge that the transposon must be widely disseminated and have reverted to this term.

For cases where carriage is confirmed, have the authors delved into the Sequence Read Archive (SRA) for associated sequence read data that might provide insights into copy number?

We are grateful for this suggestion. We asked the authors of Jackson et al, to provide details of which isolates are resistant and susceptible to amikacin and piperacillin tazobactam since raw read data were available on the SRA. They kindly provided these data and its analysis in terms of transposon copy number has added further support to our findings and increased the relevance, to include urinary isolates from the USA and CLSI breakpoints.

In the discussion, the authors highlight an important experiment that could enhance the manuscript, although it may not be deemed a major issue. Exploring copy number and its correlation with exposure to amikacin would be intriguing. Specifically, investigating the number of copies required for a transition from susceptible to non-susceptible could provide valuable insights.

We do to a large extent define copy number versus S/R status based on the population analysis in figures 4 and 5. Because of the existence of promoter mutations, laboratory selection with amikacin challenge might actually prove very complex to interpret. And there are multiple ways of increasing copy number if the transposon is plasmid encoded (replicon mutations etc). As discussed in response to referee 1, we have modified the manuscript to tone down the sense that elevated copy number is exclusively determined by IS26 amplification, for which we have no evidence.

Please ensure that all WGS data is deposited to appropriate database as soon as possible.

The data are deposited on ENA.

Reviewer #3: Introduction

Technically, only about 30% of patients with sepsis will have a BSI. Maybe use “frequently” instead and consider combining the first two setences.

Modified as requested

I feel like ARO is the common acronym for antibiotic resistant organisms (rather than ABR). Any way you can rework? Not essential.

Whilst it is always nice to hear about a new acronym, we have modified otherwise.

In many jurisdictions, “the antibiotics of choice tend” [realistically the empiric antibiotics used in a centre will be a function of the risk/proportion of ARO at that centre or in that patient population]

Agreed of course. Modified as requested

Extended spectrum beta-lactam resistance is most commonly mediated by CTX-M…. I would think TEM is more common overall (ampicillin/cefazolin)

AmpC is the most common beta-lactamase among *E. coli*, but TEM-1 is the most common mobile beta-lactamase, we would agree. This section has been modified to clarify.

When people can’t use a beta-lactam, they could use a carbapenem before an aminoglycoside [e.g., in North America] except in certain European settings.

Carbapenems are beta-lactams. But we totally follow the point and have clarified to explain that carbapenem use might be restricted more actively in some regions.

Results:

The (imperfect but important) relationship between amikacin and oxa-1 is well articulated and interesting. Is this pip-tazo resistance independent of being ceftriaxone resistant [e.g.you appear to be describing a system within the CTX-M-15 subgroup -- but if CTX-M-15 isn’t there, is there enough OXA-1 to still be piperacillin-tazobactam R like is seen with the TEM hyperproducers?]

A sub-group of isolates in our study were CTX-M-15 negative, and we showed the same finding. In our experience CTX-M-15 has no baring on piperacillin/tazobactam MIC in the presence of OXA-1. Compared with TEM-1, it is a weak piperacillin hydrolysing enzyme and even if relegated to the role of an inhibitor sink, the sink has little effect on the degree of OXA-1 inhibition seen at standard tazobactam testing concentrations (which is very little anyway).

Discussion:

The issue with MERINO was that susceptibility testing relied on an E-test which was recalled for being inaccurate. BMD testing showed that a fair number of isolates were, actually, pip-tazo resistant (particularly as breakpoints have been changed) and that the presumed mechanism for this resistance was OXA-1 co-production.

We have added an additional clarification

I agree that what you are proposing regarding using amikacin susceptibility to “screen” for the potential for OXA-1 could be interesting as a carbapenem sparing strategy but that it would need validation in other contexts.

Absolutely. And we have added some caveats at the end of the discussion

Figures:

Not essential at all -- just curious:

What would figures 4 panels C and D look like as a function of MIC? Worth exploring?

We do not have MICs for all the isolates (not even Vitek MICs), just a flag.

(Figure 5 can’t be done because you don’t have MICs, right?) -- what about as a function of zone size?

We do not have zone diameter, just a flag.

PLOS authors have the option to publish the peer review history of their article (what does this mean?). If published, this will include your full peer review and any attached files.

If you choose “no”, your identity will remain anonymous but your review may still be made public.

Do you want your identity to be public for this peer review? For information about this choice, including consent withdrawal, please see our Privacy Policy.

Reviewer #1: No

Reviewer #2: No

Reviewer #3: No
